# Transmissible ER stress shapes the leukemic microenvironment

**DOI:** 10.18632/oncotarget.27012

**Published:** 2019-06-25

**Authors:** John T. Butler, Peter Kurre

**Affiliations:** Comprehensive Bone Marrow Failure Center, Children’s Hospital of Philadelphia, Abramson Cancer Center, Perelman School of Medicine, University of Pennsylvania, Philadelphia, PA, USA

**Keywords:** AML, unfolded protein response, extracellular vesicles, BMP

Acute Myeloid Leukemia (AML) is a genetically heterogeneous disease that arises from clonal expansion of rare hematopoietic stem cells (HSC) bearing acquired somatic mutations. Specific molecular lesions define key subgroups for risk group stratification, and current therapy rapidly induces initial remissions in over 80% of patients. Yet, nearly half of these will ultimately relapse [1]. While genetic adaptation contributes to disease persistence and relapse, evidence is now exceedingly strong that the expanding AML clones effect a series of adaptive, and in part inflammatory, niche changes that protect residual AML cells from elimination by chemotherapy [2]. The development of a self-reinforcing leukemic niche in the bone marrow (BM) can therefore be considered a constitutive aspect of leukemogenesis and implies an unmet need to more fully understand how AML subverts the BM stroma toward chemo-resistance. Recent work in several hematologic and non-hematologic cancers has revealed endoplasmic reticulum (ER) stress with induction of an unfolded protein response (UPR) as one mechanism by which compartment wide chemotherapy resistance can arise [3].

## ER stress, the unfolded protein response and extracellular vesicles

ER stress results from protein misfolding in conditions of nutrient or oxygen deprivation, and engages an unfolded protein response (UPR) program to enable chaperone production and protein repair. Under excessive proteotoxic stress, the UPR pathway can also activate cell death via caspases and Bcl family members [4]. These context-dependent outcomes are triggered through the central UPR sensor gene GRP78, and differentially regulated via three downstream branches. The PERK-eIF2α branch halts protein translation, allowing protein repair before resuming protein synthesis. IRE-1/XBP1 and ATF6 branches upregulate protein chaperones through stimulation of nuclear ER stress response elements. Interestingly IRE-1/sXbp-1 axis signaling enhances survival in multiple cancer types, and is detectable in 82% of AML cell lines and 71% of AML patient samples, but not in normal CD34+ myeloid stem cells [5]. Conversely, silencing of IRE-1 and other UPR components in AML, multiple myeloma (MM) and chronic lymphocytic leukemia (CLL) mediates apoptotic or anti-tumor effects [6].

**Figure 1:**
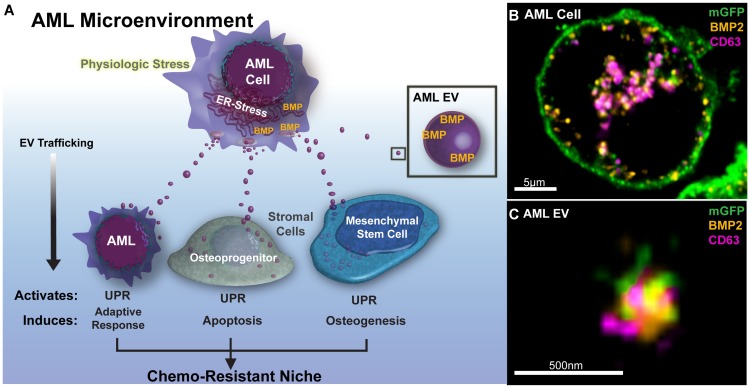
Extracellular vesicles traffic BMP2 in the AML microenvironment. (**A**) Acute Myelogenous Leukemia cells (AML) in the bone marrow microenvironment demonstrate marked ER-stress and high expression of bone morphogenic proteins. Under conditions of ER-stress, AML cells shed copious extracellular vesicles (EVs) that transmit endoplasmic reticulum (ER) stress and bone morphogenic proteins (BMP) to recipient bone marrow cells. This activates the Unfolded Protein Response Pathway (UPR) and leads to adaptive changes among stromal components in the leukemic microenvironment to enforce a chemo-protective niche (**B**) BMP (orange) expression is upregulated in AML cells (Molm-14mGFP, modified to express a myristoylated GFP tag), explanted from xenograft animals, and is compartmentalized into intracytoplasmic membranes (mGFP) along with CD63 (magenta) which are indicative of pre-exosomal multivesicular bodies. *Central slice from 3D-Airyscan Z-stack, scale bar = 5 μm*. (**C**) EVs isolated from ER-stressed Molm-14 cells co-stain with anti-BMP2 and EV-marker CD63. *Airyscan super resolution technique, scale bar = 500 nm.*

The intriguing recent discovery of transmissible ER stress (TERS) in the solid tumor microenvironment indicates that the transfer of UPR responses to bystander cells (e.g. other tumor or myeloid cells) via secreted factors can generate drug resistance in the process [7]. Pursuing this possibility in the AML niche, our recent study not only confirmed the transmission of ER stress to mesenchymal stromal cells (MSC) and osteoprogenitor cells (OPC), but revealed that the resulting phenotypic changes in MSC resulted from transfer of AML-derived extracellular vesicles (EVs) (Figure 1A) [8]. That observation extends prior work from our group and others to show the broad impact of AML EV in the BM niche, reviewed in [9].

EV biogenesis is a constitutive cellular process resulting in the release of different vesicle subclasses that traffic between cells and signal through their protein and nucleic acid cargo [9]. We observed that AML cells under ER-stress increase EV release with coincident gains in EV-associated BMP2 levels, whereas vesicle-free BMP2 in the supernatant remained unchanged. Intriguingly, both ER stress response and BMP expression in our xenograft studies rapidly subsided when AML cells were explanted for propagation in tissue culture. Those studies await independent validation in patient samples, but in additional super resolution imaging studies, we corroborated BMP2 increases in the AML cells and the colocalization with the membrane- and EV-associated tetraspanin CD63 within intracytoplasmic vacuoles, indicative of pre-exosomal multivesicular bodies (Figure 1B). EVs released from these cells also stained with BMP2 (Figure 1C). Altogether, our data suggest that EVs rich in bone morphogenic protein (BMP) -2 may serve as a mechanism for UPR transfer in the AML niche.

## A UPR perspective on compartmental chemotherapy resistance

More broadly, published work, including ours, indicate a role for ER stress in adapting the leukemic compartment. AML-EVs already serve several known protumorigenic roles in modulating bystander cells, including stromal cells, hematopoietic progenitors and NK cells, and contribute to drug resistance in myeloid malignancies, including AML [9, 10]. The notion of BMP2 specifically as a responsible TERS factor in the AML BM is consistent with its known role in leukemia progression and induction of osteogenic differentiation, respectively [11, 12]. Whether EVs serve as carriers of UPR inducing protein cargo more broadly awaits further investigation. Furthermore, a link between ER-stress and drug resistance is already well supported, but mechanistic insight into the precise relationship is currently missing [13]. Similarly, whether the UPR signaling pathways activate and sustain chronic inflammation in the AML niche, and the role of EV in reprogramming the microenvironment toward drug resistance remains a pivotal question to be clarified, Table 1.

**Table 1:** UPR and drug resistance; open questions

• What is the role of EV in UPR transfer more broadly
• Do AML EVs selectively target specific recipient cells within the microenvironment
• What is the critical AML EV cargo beyond BMP2 (protein, RNA)
• What is the molecular target or signaling pathway that translates UPR to drug resistance
• Are targets leukemia-subtype, or even patient-specific
• What is the relationship between UPR and inflammation in the BM

The genetic heterogeneity of AML combined with its dynamic clonal succession and adaptive niche remodeling provide a formidable therapeutic challenge [1]. Understanding the sanctuary function of the BM in AML will enable us to develop new adjuvant therapies without further escalating treatment toxicity Insight into the role of the transmissible UPR promises to reveal additional unexplored targets to overcome extrinsic chemoprotection.
